# The effects of vitamin D supplementation on interictal serum levels of calcitonin gene-related peptide (CGRP) in episodic migraine patients: post hoc analysis of a randomized double-blind placebo-controlled trial

**DOI:** 10.1186/s10194-020-01090-w

**Published:** 2020-02-24

**Authors:** Zeinab Ghorbani, Pegah Rafiee, Akbar Fotouhi, Samane Haghighi, Reyhaneh Rasekh Magham, Zeynab Sadat Ahmadi, Mahmoud Djalali, Mahnaz Zareei, Soodeh Razeghi Jahromi, Sahar Shahemi, Maryam Mahmoudi, Mansoureh Togha

**Affiliations:** 1 Department of Cellular and Molecular Nutrition, School of Nutritional Sciences and Dietetics, Tehran University of Medical Sciences, Tehran, Iran; 2 Student Research Committee, Department and Faculty of Nutrition Sciences and Food Technology, Shahid Beheshti University of Medical Sciences, Tehran, Iran; 3 Department of Epidemiology and Biostatistics, School of Public Health, Tehran University of Medical Sciences, Tehran, Iran; 4 Headache Department, Iranian Center of Neurological Research, Neuroscience Institute, Tehran University of Medical Sciences, Tehran, Iran; 5 Department of Nutrition, Faculty of Medical Sciences, Science and Research Branch, Islamic Azad University, Tehran, Iran; 6 Department of Nutrition, School of Health, Iran University of Medical Sciences, Tehran, Iran; 7 Department of Clinical Nutrition and Dietetics, Faculty of Nutrition and Food Technology, Shahid Beheshti University of Medical Sciences, Tehran, Iran; 8 Nutrition and Endocrine Research Center, Research Institute for Endocrine Sciences, Department of Clinical Nutrition and Dietetics, Shahid Beheshti University of Medical Sciences, Tehran, Iran; 9 Pediatric Gastroenterology and Hepatology Research Center, Children’s Medical Center, Tehran University of Medical Sciences, Tehran, Iran; 10 Dietitians and Nutrition Experts Team (DiNET), Universal Scientific Education and Research Network (USERN), Tehran, Iran; 11 Headache Department, Neurology Ward, Sina University Hospital, School of Medicine, Tehran University of Medical Sciences, Tehran, Iran

**Keywords:** Cholecalciferol, Disability, Headache, CGRP, Migraine with Aura

## Abstract

**Background:**

Emerging evidence showed promising effects of vitamin D on headaches characteristics. Thus, it seems there is still a need for more researches to clarify the mechanisms by which this vitamin exerts anti-migraine effects.

**Methods:**

The present study was conducted as a 16-week randomized double-blind placebo-controlled trial on 80 episodic migraine patients allocated in 2 parallel groups each consisted of 40 patients who received vitamin D 2000 IU/d or placebo. At baseline and after the intervention completion, headache diaries and migraine disability assessment questionnaire (MIDAS) were used to assess migraine related variables in patients. Also, interictal serum concentration of calcitonin gene-related peptide (CGRP) (as the dominant mediator of migraine pain pathogenesis) was evaluated using ELISA method.

**Results:**

The mean (SD) of age in the vitamin D and placebo groups was 37 (8) and 38 (12) years, respectively. ANCOVA test adjusted for baseline values, and confounders showed vitamin D supplementation resulted in a significant improvement in MIDAS score after 12 weeks in the intervention group (21.49 (16.22–26.77)) compared to placebo (31.16 (25.51–36.82) *P* value: 0.016). Moreover, after controlling for baseline levels, and other variables using ANCOVA, CGRP level was appeared to be significantly lower following vitamin D supplementation (153.26 (133.03–173.49) ng/L) than the patients in the placebo arm (188.35 (167.15–209.54) ng/L) (*P* value = 0.022).

**Conclusion:**

According to the current findings, vitamin D supplementation in episodic migraineurs, particularly in those with migraine with aura, may potentially improve migraine headache characteristics and disability probably through attenuating CGRP levels. Therefore, these results could provide a new insight into anti-nociceptive effects of vitamin D; however, more studies are required to confirm our findings.

**Trial registration:**

The trial is registered in the Iranian registry of clinical trials (IRCT) at 11 July 2018, with IRCT code: IRCT20151128025267N6.

## Background

According to the report of global burden of disease (GBD) 2016, migraine headache has been established as the leading disability cause in the globe among under 50 years old [1]. It has been estimated that migraine affects about 11% of adults with a major impact on those aged between 25 to 55 years [1, 2], which consequently lead to the high headache burden on individuals, communities, health care systems, and societies [3]. Migraine is 2–3 times more common in women than in men. Migraine attacks are usually more prolonged, with higher intensity and more disability among females [4].

Two main categories of migraine include episodic migraine (having < 15 headache days per month) and chronic migraine (having ≥15 headache days per month, of which at least 8 days are with migraine features or response to Triptans, for at least 3 months) [5]. If patients who suffer from episodic migraine, especially those with frequent attacks, are not treated appropriately, it can lead to chronic type which is reported to be more severe with higher disability [6, 7].

Both genetics and environment are supposed to be related to migraine development. Some dietary factors, misuse of caffeine, hormonal fluctuations, high stress level, increased weight, smoking, and suffering from tension type headache or medication overuse headache are among the suggested factors affecting migraine susceptibility and or chronification [8].

There is no established mechanism to explain migraine pathogenesis, although there have been much efforts directed in this field. The current evidence suggest a variety of mechanisms which are thought to be involved in pathophysiology of migraine including cortical spreading depression (CSD), trigeminovascular pathway activation (a pathway for nociceptive information conduction), neuro-inflammation as well as dysfunction in vascular system [9]. Also, different neuropeptides such as calcitonin gene-related peptide (CGRP), and pituitary adenylate cyclase-activating peptide (PACAP) with vasoactive properties are believed to be released during migraine attacks. The release of these factors could affect activation of trigeminovascular system and subsequently might induce mast cells degranulation, vasodilatation of arteries, and plasma leakage, which all are supposed to be involved in migraine attacks initiation [9].

Current evidence highlighted a need for compounds that suppress the neuro-inflammatory state in migraine as novel effective medications in its treatment [8, 10, 11]. Keeping this in mind, adverse effects that related to the present migraine pharmacological treatments (including weight reduction or weight gain, fatigue/ sleepiness, sweeting, night dreams, gastrointestinal upset, hypotension or reduced concentration) might negatively impact on the patients’ adherence to drug therapy, and therefore reduce the efficacy of treatment procedure [12–15]. Hence, applying novel therapeutic approaches including nutritional agents could be promising strategies that can favorably modify migraine characteristics and its associated disability [8]. Magnesium, omega 3, melatonin, coenzymes Q10, riboflavin and folate are among the most studied dietary supplements that have been suggested to be beneficial in prophylaxis of migraine [16, 17]. Also, vitamin D is one of the dietary agents that has been taken into consideration for pain and migraine improvement [18–23]. In this regard, our team has recently published a review paper aiming to address the association between vitamin D and headache [8]. It was estimated that about 45–100% of migraine or headache patients might suffer from deficiency/insufficiency of vitamin D. It was further shown that there seems to be a negative correlation between serum vitamin D concentrations and headaches frequency [8]. Also, in our previous paper [24], we showed that supplementation with vitamin D (2000 IU/d) could reduce number of headache attacks, decrease the need for analgesic consumption and attenuate intensity and duration of headaches. We also observed that this vitamin might suppress neuro-inflammation in migraineurs through lowering the levels of inducible nitric oxide synthase (iNOS) and probably interleukin (IL)-6 [24]. However, it seems there is still a need to further clarify the mechanisms by which vitamin D exerts anti-migraine effects. Therefore, in the same study, we aimed to explore changes in interictal levels of the dominant mediator of migraine pain pathogenesis [25] (serum CGRP) following prophylactic administration of vitamin D. In addition, vitamin D effects on migraine associated disability was aimed to be determined.

## Methods

### Study design

The details on study design and data collection has been described in our previous publication [24]. Briefly, the present study was conducted as a 16-week (12-week intervention following a 4-week baseline period) randomized double-blind placebo-controlled trial on 80 episodic migraineurs aged between 18 and 45 years to investigate the effects of 2000 IU/d vitamin D supplementation on headache characteristics and CGRP levels compared with placebo.

### Participants and settings

The current randomized clinical trial (RCT) was performed in Tehran, Iran from July 2018 to July 2019. Totally, 98 migraine patients who were referred to the tertiary headache clinic of Sina University Hospital, were interviewed for the current trial. Inclusion criteria were diagnosis of episodic migraine (EM) according to headache characteristics and the International Classification of Headache Disorders, 3rd edition (ICHDIII criteria) [5], age between 18 to 45 years, having body mass index (BMI) between 18.5–30 kg/m2 and suffering from migraine for at least 6 months prior to study. Exclusion criteria were unwillingness to participate in the study, suffering from medication overuse headache in 3 months prior to study, taking vitamin D supplements in 3 months prior to study, taking magnesium, calcium, zinc, vitamin B groups or vitamin C supplements during the study period, taking anti-epileptic drugs such as topiramate, sodium valproate and carbamazepine, taking thiazide diuretics, glucocorticoid, statins or orlistat, taking anti-psychotic drugs. Also, menopause, pregnancy and lactation, and suffering from gastrointestinal disorders, liver and kidney disorders, cancer, sarcoidosis, rickets, and osteomalacia according to physician diagnosis and/or past medical history were among the other exclusion criteria.

### Participants’ randomization and blinding

**Fig. 1:**
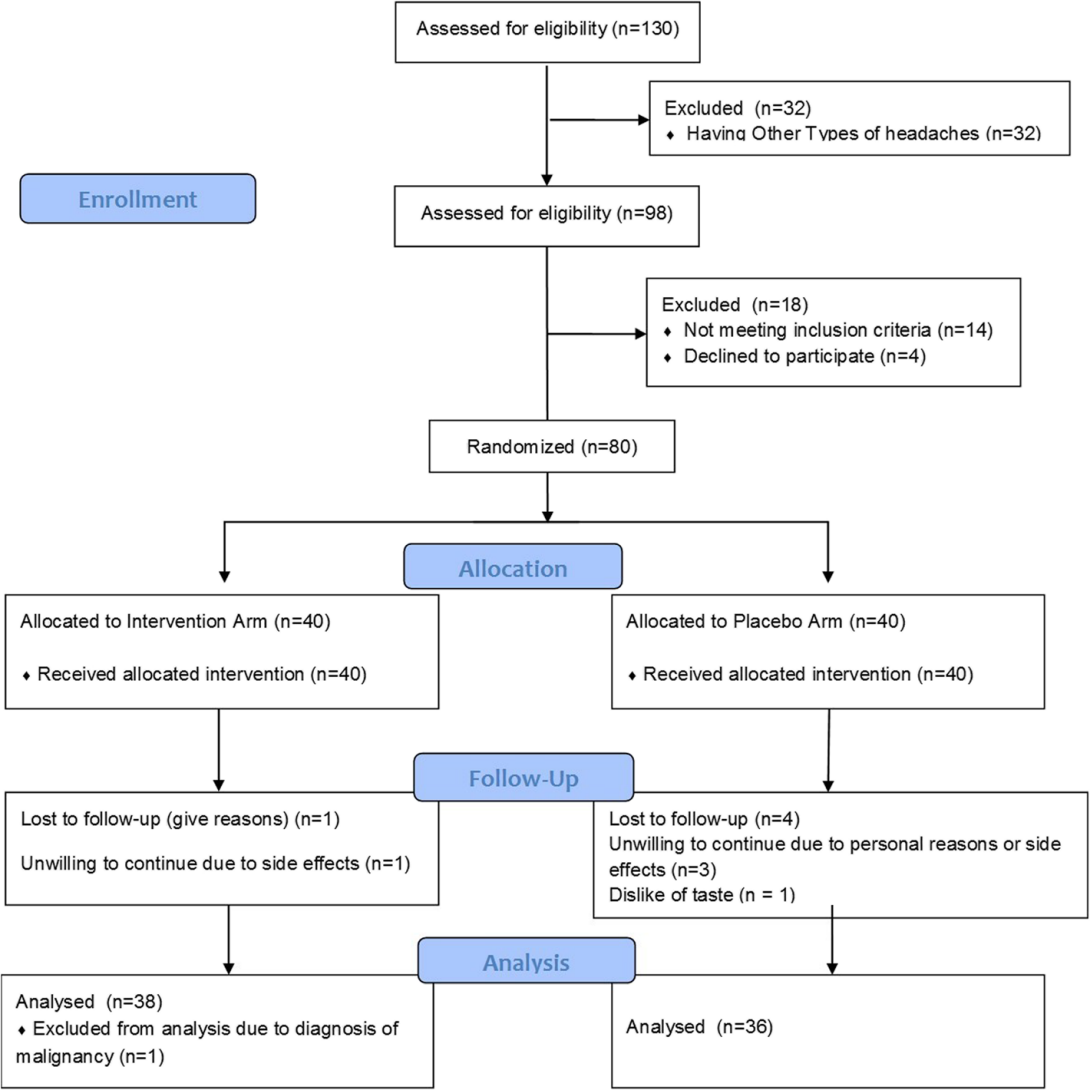
Flow-Diagram of Studied Patients

After initial examination by the study expert headache-specialist neurologists, 32 subjects were excluded due to exclusion criteria as illustrated in Fig. 1. From the beginning of the study, A (vitamin D supplement) and B (placebo) codes were available to the researchers to recruit the patients. Only one of the staff of headache department was aware of codes. Block randomization was applied to ensure that both participants and researchers were blind to treatment allocation. Thus, 80 patients who fulfilled the inclusion criteria were randomly allocated in a 1:1 ratio in blocks of 4 to receive either A or B supplements and stratified based on gender. Since assignment of patients was concealed until statistical analysis completion, the participants, neurologists, and investigators were blinded to the group allocation throughout the trial.

### Data collection

At the first visit, all demographic and socioeconomic information including age, sex, marital status, educational status, and job, headache onset years, having migraine with/without aura, familial history of migraine and smoking status, number and type of medications, and data on past medical history of patients were collected through face to face interview by an expert researcher.

Anthropometric data including weight, and height were also evaluated at study entry and the end of the trial as follow: body weight was assessed to the nearest 0.5 kg and height to the nearest 0.1 cm applying a Seca 755 dial column medical scale and standard stadiometer, respectively. BMI was calculated as weight in kilograms divided by height in square meters.

### Intervention

Patients in the intervention group received 1 pearl containing 2000 IU of vitamin D/d in addition to routine medications prescribed by our study neurologists. The patients in the placebo group were given 1 placebo pearl which was indistinguishable from vitamin D pearls in taste and appearance. Throughout the trial, the subjects were allowed to consume their usual acute/ prophylactic drugs, though they were asked not to change their medications during the trial. Both vitamin D and placebo pearls were produced by Zahravi Pharmaceutical Company, Tabriz, Iran.

### Outcome measures

#### Headache diaries assessment

All participants received a 30-days headache diary to fill out the migraine characteristics (designed by the senior researcher, Prf. M.T and has been described in details previously) [26]. Briefly, the participants were asked to complete their headache diaries during a month prior to beginning the intervention and also the last month of trial. Detailed results of present RCT on headache characteristics based on these diaries have been reported previously [24]. To better understand current findings, we only mentioned the results on the number of headache days per month.

In addition, at the end of investigation we asked the participants to roughly rate their overall perception of headaches improvement following the current intervention using a six-point Likert scale, ranging from 1 (less than 10%) to 6 (more than 70%) [27].

#### Migraine disability assessment questionnaire (MIDAS)

At baseline and after the intervention completion, MIDAS questionnaire was used to assess migraine related disability in patients. The questionnaire includes seven questions that examine the levels of impact of headaches on work, home, social and leisure activities over the past 3 months. Questions 1 through 5 of this questionnaire assess the migraine-related decline in performance. The questionnaire was validated previously in Iran [28].

#### Blood collection and serum analysis

At the first visit and the end of the trial, 5 ml blood samples were obtained from all the patients to assess serum concentration of 25OHD and CGRP. Blood samples were centrifuged at 4 °C for 10 min and serum samples were kept at − 80 °C until laboratory analysis. Vitamin D levels in the form of serum 25OHD (S25OHD) were evaluated using chemiluminescence immunoassays (CLIA) method. Serum level of CGRP was evaluated using enzyme linked immunosorbent assay (ELIZA) method and Crystal Day kit, E1061 Hu, 96 tests (Crystal day, China). The interval of time from the last attack of headache to the day of blood samples collection was also recorded.

#### Compliance, adverse events and patients’ satisfaction score

Compliance was indicated using tablet count after 12-week of intervention. A studied participant was considered compliant if ≥70% of the tablets had been used throughout the trial. During study follow-up, the compliance was additionally stimulated by weekly telephone calls and reminding the participants to use the supplements. Also, after 12-week trial, S25OHD status was evaluated in order to assess the compliance of the subjects in the intervention arm. Also, patients were asked to report any minor or major adverse effects which were likely to be associated with supplementation. The subjects were also asked to rate their satisfaction with participation in the study using a five-point Likert scale ranging from 1 (very dissatisfied) to 5 (very satisfied) [27].

### Sample size calculation and statistical analysis

According to the below formula, it was assumed that by estimating 40 samples in each group with an average of 8 headache day/month (standard deviation (SD): 4 days), aiming to detect at least 4 days reduction in headache days, 90% statistical power will be achieved (α = 0.05, β = 0.90, and S = 4 and d = 4) [19].

$$ n=2\frac{{\left(Z1-\frac{\alpha }{2}+Z1-\beta \right)}^2{S}_2}{(d)^2} $$

Means (SD) were used to describe the quantitative variables and frequencies (percentage, %) were reported for categorical variables. Within and between groups comparisons were made using paired sample t-test or either independent t test or Chi square test, respectively. Further, one-way analysis of variance (ANOVA) was used to compare between groups when aiming to assess variables changes before and after supplementation with vitamin D or placebo in episodic migraine patients with/without aura. In order to analysis the effects of intervention on headache characteristics, MIDAS score, and serum CGRP levels, analysis of covariance (ANCOVA) adjusted for baseline values and confounders was applied. In the case of using ANCOVA, the adjusted means and 95% confidence intervals (95% CI) were also reported. Pearson correlation test was used to determine the correlation between changes in S25OHD and outcome variables. The figures was prepared by GraphPad Prism 5.0 (GraphPad Software, Inc.). Significance level was defined as *P* < 0.05 level. All statistical analyses were performed using SPSS program version 19 (IBM Corporation, New York, USA).

## Results

### Baseline, clinical and anthropometric characteristics

Figure 1 displays the studied subjects flow-diagram. Initially, 130 headache patients were examined by our study headache-specialists of whom 32 had other types of headache concurrent with migraine including cervicogenic headache, or secondary headaches. Thus 98 migraineurs were assessed for eligibility criteria of whom 80 were included in the trial; and randomized into vitamin D (*n* = 40, 80.0% women) or placebo group (*n* = 40, 80.0% women). Four subjects in the placebo group and 2 patients in the intervention arm were lost to follow-up. The reasons are described in Fig. 1.

Table 1 displays the baseline characteristics, and clinical and anthropometric data of the studied participants. The two studied groups did not differ significantly in age, sex, headache onset years, socio-economic status, having migraine with/without aura, familial history of migraine or smoking status. The mean (SD) of age in the vitamin D and placebo groups was 37 (8) and 38 (12) years, respectively. On average, headache onset years in the patients in the intervention and control group were about 12 and 11 years, respectively. Additionally, approximately 32.5% of patients who received vitamin D supplements and 25.0% of the placebo taking patients were suffering from migraine with aura. The mean BMI was approximately around 25.83 kg/m2 in vitamin D group, and 25.36 kg/m2 in placebo group before and after the study, with no significant differences between groups.

**Table 1:** Baseline demographic, anthropometric and clinical data of episodic migraine patients in a randomized controlled trial of vitamin D vs. placebo

	Vitamin D (*n* = 40)	Placebo (*n* = 40)	*P* value^#^
Age (year)	37 (8)	38 (12)	0.819
Headache Onset years (year)	12 (8)	11 (8)	0.679
Body mass index (kg/m2)	25.83 (4.07)	25.36 (4.29)	0.613
Sex			
Female	32 (80.0%)	32 (80.0%)	1.000
Male	8 (20.0%)	8 (20.0%)	
Marital Status			
Married	31 (77.5%)	26 (65.0%)	0.339
Single	9 (22.5%)	13 (32.5%)	
Widow/widower	0 (0%)	1 (2.5%)	
Educational Status			
High school	13 (32.5%)	16 (40.0%)	0.293
BSc	11 (27.5%)	7 (17.5%)	
MS	8 (20.0%)	13 (32.5%)	
PhD and higher	8 (20.0%)	4 (10.0%)	
Job			
No job/house wife	12 (30.0%)	16 (40.0%)	0.480
Student	1 (2.5%)	3 (7.5%)	
Employee	23 (57.5%)	17 (42.5%)	
Self employed	4 (10.0%)	4 (10.0%)	
Migraine Aura			
Migraine with aura	13 (32.5%)	10 (25.0%)	0.459
Migraine without aura	27 (67.5%)	30 (75.0%)	
Familial History of Migraine			
Yes	26 (65.0%)	32 (80.0%)	0.133
No	14 (35.0%)	8 (20.0%)	
Smoking Status			
No	37 (92.5%)	35 (87.5%)	0.549
Yes	0 (0%)	1 (2.5%)	
More than 5 cigarette per week	3 (7.5%)	4 (10.0%)	
Less than 5 cigarette per week	0 (.0%)	0 (0%)	

Values are expressed as mean (SD) or number (%) as appropriate

^#^Using either Independent t test or Chi square according to the type of data

### Serum vitamin D status

Mean baseline S25OHD levels among patients received placebo was significantly higher than those in the vitamin D group (*P* value = 0.017); however, after 12 weeks of study, serum levels of this vitamin significantly increased in the vitamin D group (from 27.31 to 39.65 ng/ml; *P* value< 0.001) compared to no significant changes observed in the placebo group (from 33.70 to 33.95 ng/ml) (*P* value for between group comparison = 0.062).

### Medications consumption

**Fig. 2:**
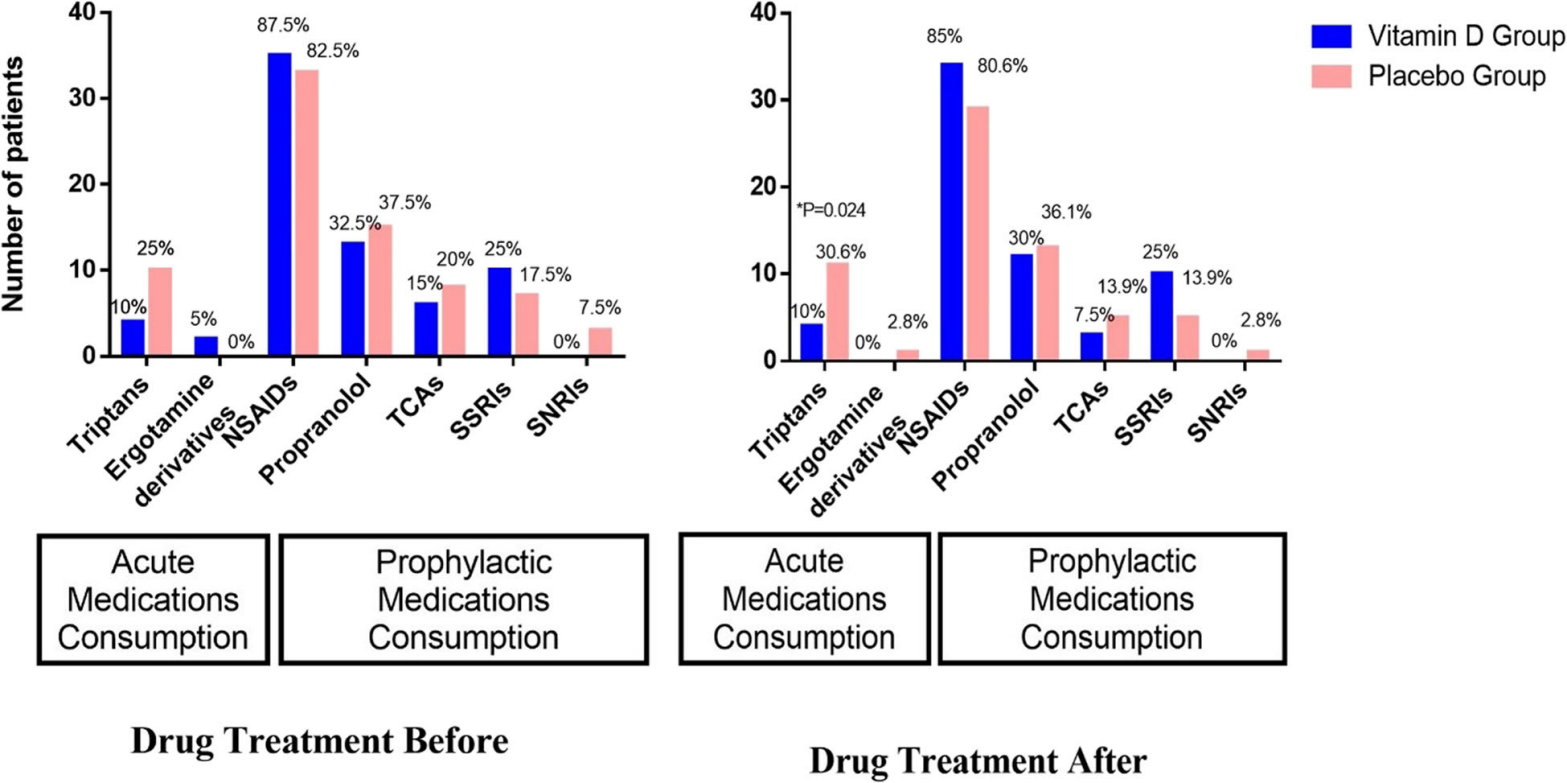
Types of Medications Used By Studied Patients Throughout The Trial. NSAIDs, Nonsteroidal anti-inflammatory drugs. TCAs, Tricyclic antidepressants. SSRIs, Selective serotonin reuptake inhibitors. SNRIs, Serotonin-Norepinephrine Reuptake inhibitors

The medications consumption of both studied groups at baseline and after the intervention consisted of abortive and prophylactic drugs are demonstrated in Fig. 2. There was not any significant differences between groups before and after the trial on type of abortive/prophylactic medications use, except for using triptans in the placebo group which was significantly higher than that of vitamin D group after the trial (*P* = 0.024).

### The number of headache days and frequency of attacks

Detailed results of present RCT on headache characteristics have been reported previously [24]. Patients in treatment and placebo group were estimated to experience about 8.04 and 7.69 headache days per month, respectively. No significant differences were noted regarding baseline values of number of headache days between the groups. Paired t-test revealed statistically significant reduction in the number of headache days among the patients who received vitamin D supplements (mean changes: − 2.90 (3.03); *P* value< 0.001), whereas there were no significant changes observed within the placebo group when comparing to the baseline data. Following the trial, ANCOVA test adjusted for baseline values and confounders demonstrated that patients in vitamin D group had significantly lower headache days (4.71 day/month) in comparison with placebo group (6.43 day/month) (*P* value from ANCOVA test =0.031).

### Migraine disability

There were no significant differences in baseline MIDAS scores in vitamin D and placebo groups (mean values = 29.75 and 36.87, respectively). Vitamin D supplementation resulted in a significant improvement in MIDAS score after 12 weeks in the intervention group (21.49 (16.22–26.77)) compared to placebo (31.16 (25.51–36.82) *P* value from ANCOVA test = 0.016)). Within group comparisons revealed this score significantly reduced in vitamin D group (mean changes: − 10.38 (12.29); *P* value< 0.001), while there were no significant changes noted within the placebo group (Table 2).

**Table 2:** Changes in migraine characteristics, the use of analgesics and serum CGRP levels before and after supplementation with vitamin D or placebo in episodic migraine patients

	Vitamin D Group (*n* = 38)		Placebo Group (*n* = 36)		*P* value for baseline comparison	*P* value for end of trial comparison
	Baseline	After 12 Weeks	Baseline	After 12 Weeks		
Serum 25OHD Levels (ng/ml)						
Mean (SD)	27.31 (12.30)	39.65 (13.45)	33.70 (11.08)	33.95 (12.14)	0.017	0.062^£^
Changes from baseline	12.34 (6.34)		−0.28 (5.90)			
*P* value for within-group comparison	< 0.001^#^		0.782^#^			
Number of Headache Days per month						
Mean (SD)	8.04 (3.35)	5.07 (2. 82)	7.69 (3.39)	7.26 (3. 86)	0.644	0.006^£^
Adjusted mean (95%CI)		4.71 (3.64–5.77)		6.43 (5.28–7.58)		0.031 ^δ^
Changes from baseline	−2.90 (3.03)		- 0.14 (2.91)			
*P* value for within-group comparison	< 0.001^#^		0.776^#^			
Migraine Related Disability (MIDAS score)						
	29.75 (18. 54)	19.38 (16. 43)	36.87 (21. 55)	35.36 (19. 45)	0.117	< 0.001^£^
Adjusted mean (95%CI)		21.49 (16.22–26.77)		31.16 (25.51–36.82)		0.016 ^δ^
Changes from baseline	−10.38 (12.29)		0.33 (14. 82)			
*P* value for within-group comparison	< 0.001^#^		0.893^#^			
Serum CGRP levels (ng/L)						
Mean (SD)	195.27 (78.45)	167.961 (80.112)	166.05 (58.32)	176.71 (72.22)	0.063	0.626^£^
Adjusted mean (95%CI)		153.26 (133.03–173.49)		188.35 (167.15–209.54)		0.022 ^€^
Changes from baseline	−27.31 (59.69)		12.57 (23.85)			
*P* value for within-group comparison	0.006^#^		0.004^#^			

*P* value < 0.05 was considered significant

The second row of this table was previously published in [24] in a slightly different format

*BMI* Body mass index, *MIDAS* Migraine disability assessment questionnaire, *CGRP* Calcitonin gene-related peptide

^#^Paired sample t-test

^£^ Independent t-test

^δ^ANCOVA test adjusted for baseline values, age, sex, and BMI change

^€^ANCOVA test adjusted for baseline CGRP levels, age, sex, BMI change, the years having headache, and time from last attack

### Comparison between episodic migraine patients with or without aura

Following 12 weeks of intervention, number of headache days and disability score significantly decreased in vitamin D treated migraine patients either those with aura or without aura (*P* value≤0.002). Interestingly, migraineurs with aura who received vitamin D supplements had the highest reduction of mean frequency of headache days per month (− 4.58) and MIDAS score (− 16.92) as compared to other studied groups (*P* value≤0.001) (Table 3).

**Table 3:** Changes in number of headache days, and migraine disability scores before and after supplementation with vitamin D or placebo in episodic migraine patients with/without aura

	Study sub-groups				*P* value^*^
	Patients with migraine with aura		Patients with migraine without aura		
	Vitamin D	Placebo	Vitamin D	Placebo	
Number of Headache Days per month					
Baseline	10.58 (3.67) ^a,b^	7.75 (3.86)	6.81 (2.43) ^a^	7.67 (3.29) ^b^	0.008
After the trial	6.00 (3.45)	7.50 (3.32)	4.63 (2.40) ^a^	7.17 (4.11) ^a^	0.029
Changes	−4.58 (3.76) ^a,b^	−0.25 (3.05) ^a^	−2.09 (2.27)	− 0.10 (2.91) ^b^	0.000
*P* value^#^	0.001	0.801	< 0.001	0.868	
Migraine Related Disability (MIDAS score)					
Baseline	40.00 (26.38)	37.90 (18.53)	24.81 (10.80)	36.53 (22.75)	0.057
After the trial	23.08 (24.42)	42.90 (25.15) ^a^	17.59 (10.94) ^a.b^	32.46 (16.44) ^b^	0.001
Changes	−16.92 (15.39) ^a,b^	5.00 (20.18) ^a^	−7.22 (9.25)	−1.46 (12.20) ^b^	0.001
*P* value^#^	0.002	0.453	< 0.001	0.547	

Data are presented as mean (standard deviation)

Alphabets represent significant differences between each variable and two other variables, calculated by Bonferroni test (post-hoc)

*One-way analysis of variance (ANOVA)

# Paired sample t-test

### Patients’ overall perception of headaches improvement

**Fig. 3:**
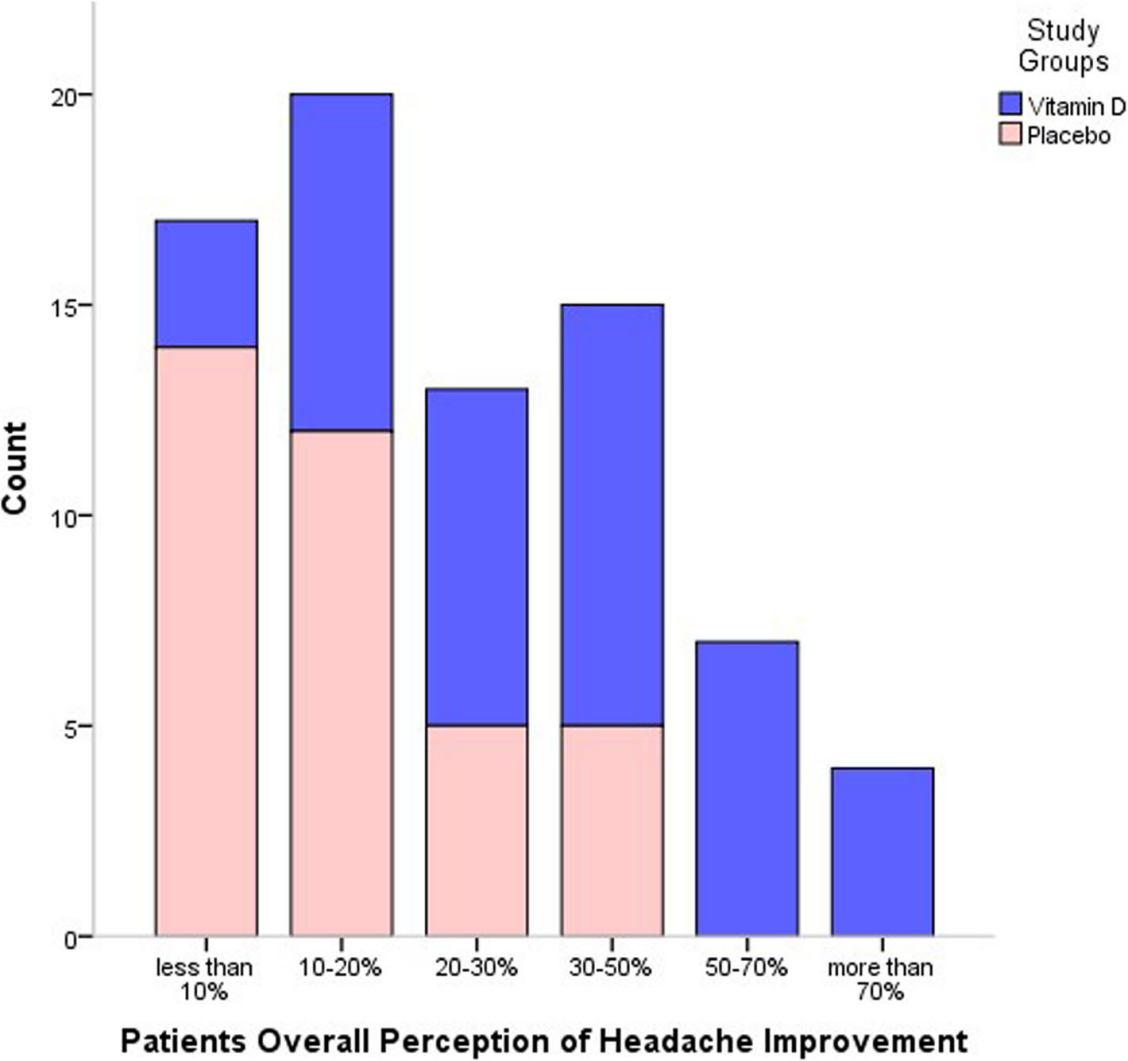
Patients Overall Perception of Headache Improvement Following 12-week Trial. (*P* value< 0.001)

About 17.5% (*n* = 7) and 10% (*n* = 4) of patients in the vitamin D group reported 50–70% and > 70% overall improvement in their headaches, respectively; while majority of patients in control group reported < 20% improvement (*P* value < 0.001) (Fig. 3).

### Serum analysis of CGRP levels

At baseline, mean time from the last attack to the day of blood samples collection was reported to be about 4 days in both vitamin D and placebo groups. At the end of trial, mean days since the last attack was estimated as 5 days in vitamin D group and 4 days in placebo group. The changes in serum concentrations of CGRP in two studied groups before and after supplementation with vitamin D or placebo are demonstrated in Table 2. At baseline, patients in vitamin D group had a slightly higher serum levels of CGRP (mean values = 195.27 and166.05 ng/L, respectively; *P* value = 0.063). Within group comparisons showed CGRP levels significantly reduced in the vitamin D group compare to baseline (mean changes: − 27.31 (59.69); *P* value = 0.006), while it slightly increased in the placebo group (mean changes: 12.57 ± 23.85); *P* value = 0.004). After the trial, the results of independent t test showed no significant differences in mean CGRP concentration of the two studied group; however, after controlling for baseline CGRP levels, age, sex, BMI change, the years having headache, and time from last attack using ANCOVA, CGRP level was appeared to be significantly lower following vitamin D supplementation among patients in the intervention group (153.26 (133.03–173.49) ng/L) than the patients in the placebo arm (188.35 (167.15–209.54) ng/L (*P* value: 0.022)).

### Correlation between changes in S25OHD and headache related variable

**Fig. 4:**
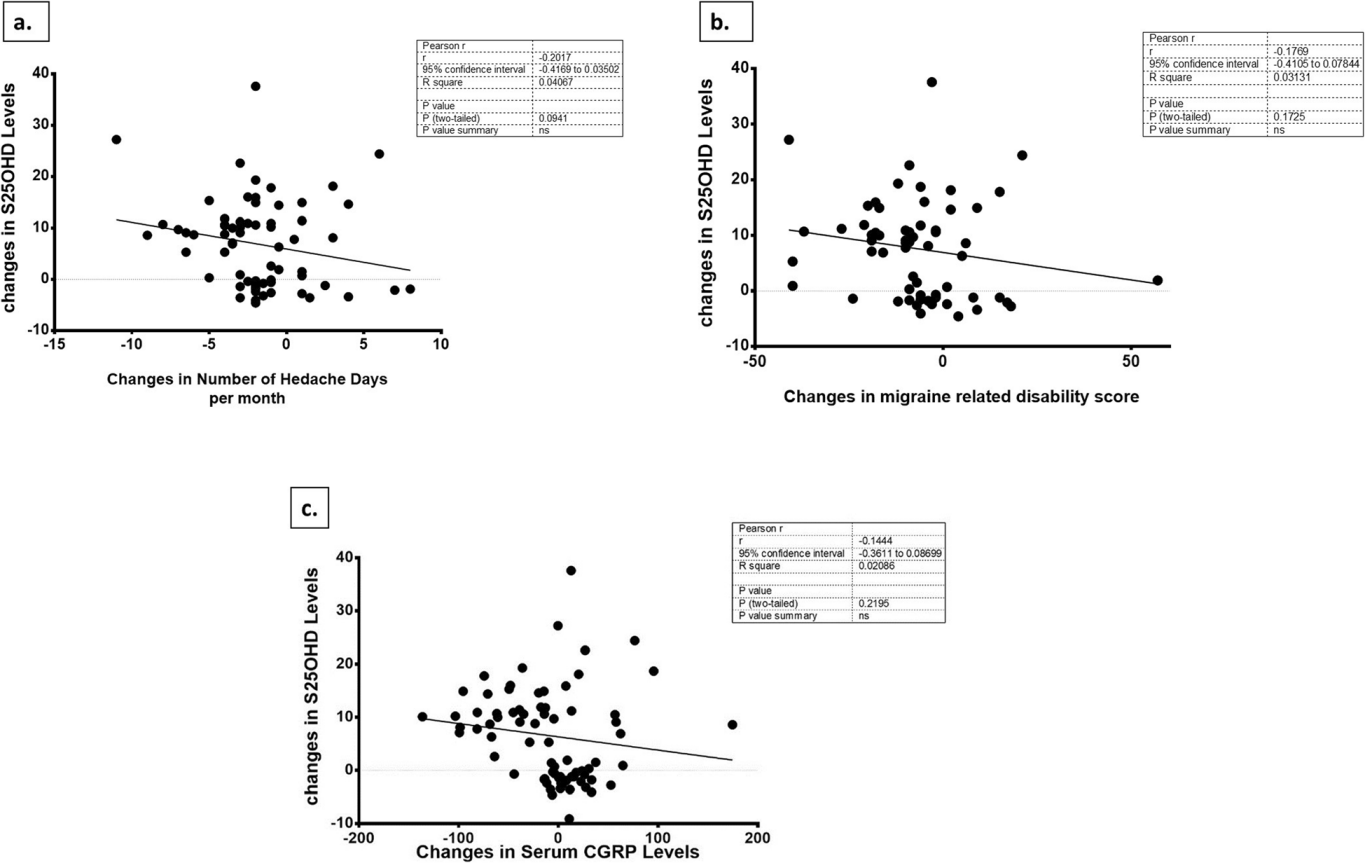
**a** The Correlation between changes in serum 25OHD and number of headache days per month throughout 12-week trial. **b** The Correlation between changes in serum 25OHD and migraine related disability score (MIDAS, migraine Disability Assessment score) throughout 12-week trial. **c** The Correlation between changes in serum 25OHD and CGRP (calcitonin gene-related peptide) levels throughout 12-week trial

Figure 4 illustrates the Pearson correlation analysis between S25OHD changes and changes in the number of headache days (Fig. 4a), changes in migraine disability score (Fig. 4b) CGRP levels changes (Fig. 4c) over the 3-month supplementation with 2000 IU/d vitamin D or placebo. Non-significant negative correlations were detected between changes in S25OHD and changes in number of headache days per month (*r* = − 0.201, *P* value = 0.094), MIDAS score (*r* = − 0.176, *P* value = 0.172), and CGRP levels (*r* = − 0.144, *P* value = 0.219) throughout the intervention (Fig. 4).

### Adverse effects and patients’ satisfaction

No statistically significant differences were observed between the studied groups with regard to the total number of patients experienced adverse effects over the trial. Minor gastrointestinal complaints were the most reported adverse effects among both groups with 5 patients (12.5%) in vitamin D group and 8 patients (22.2%) in controls. None of the studied subjects reported any serious adverse reactions, and the interventions overly were well-tolerated. The median score of patients’ satisfaction with the trial was higher among vitamin D receiving patients (median = 3) than placebo groups (median = 2) (*P* value: 0.003).

## Discussion

In our previous report of present RCT, it was first demonstrated that 12-week supplementation with vitamin D 2000 IU/d resulted in significantly lower headache days, and attacks frequency [24]. Further analysis in current study, showed that following adjustment for confounding factors (i.e. baseline disability level, sex, age, and BMI change) using ANCOVA, vitamin D is shown to significantly decrease migraine related disability in comparison with placebo group. Interestingly, these results were especially pronounced among those patients who had migraine with aura. Current study may be considered a further effort in clarifying the mechanisms through which vitamin D might be effective in migraine prophylaxis. In this regard, it is demonstrated that following the trial, mean interictal level of CGRP in patients who received 2000 IU/d of vitamin D was significantly lower than that of placebo receiving group.

These results tie well with most of the previous studies wherein vitamin D supplementation effects were investigated in relation to pain, headache or migraine [8, 29–33]. According to the recently published review article, there have been numerous studies that reported a positive linkage between vitamin D deficiency and pain associated conditions; however, no causal relationship has been noted yet [8]. There are also clinical studies that have found vitamin D supplementation may result in pain relieving effects particularly among the subjects who had S25OHD concentration less than 30 nmol/L [29–33].

There are several likely mechanisms that may explain the protective effects of vitamin D on migraine characteristics and associated disability. First, according to current results, administration of this vitamin significantly reduced serum CGRP levels, the dominant mediator [25] of migraine pain. To our knowledge current study results regarding the effects of vitamin D on CGRP are novel, therefore we cannot directly compare them with previous studies.

CGRP is a vasoactive neuropeptide containing 37 amino acids that plays a role in vasodilation, modulating immune system, neuronal inflammation and transmission of pain signals. Two main isoforms of CGRP are alpha-CGRP (which is certainly found in both peripheral and central nervous system (PNS and CNS)) and beta-CGRP (which is mainly present in enteric nervous system), only differ in 3 amino acid constituents [34]. The sensory nerve fibers that contain CGRP are particularly expressed in neuronal and vascular tissues of the cerebrum, specifically in the trigeminal ganglion. This neuropeptide could induce direct vasodilatation (independent of endothelium) through binding to the receptors (CGRP-1, CGRP-2, CGRP-3) and stimulating the release of adenyl cyclase and cyclic adenosine monophosphate (cAMP). It is thought that serotonergic system regulate CGRP secretion while nerve growth factor and NO affect its production. Additionally, CGRP is highly interacted with substance P (SP) such that both neuropeptides could usually be found in the same sites of nervous system [25, 34, 35]. As one of the most studied factors in relation to migraine, CGRP is contributed to a variety of pathophysiological mechanisms involved in pain sensitization in this type of headache. Inducing dilation in the blood vessels of dura matter, causing degranulation of mast cells and above all, playing a pivotal role in activation of trigeminovascular system which is responsible for nociceptive information conduction from the meninges to the cortex and central region of the brain, are among the suggested mechanisms [34].

The CGRP concentration is detected to be augmented in migraineurs especially during head pain periods [34, 36]. These evidences can further confirm the hypothesis of recognizing migraine as a neuro-vascular disorder and also support the potent role of CGRP and neural events that cause dilation in blood vessels in migraine pathogenesis [34, 36]. In most of the subjects who had a history of migraine, a delayed migraine attack occurs following CGRP administration in IV form; while CGRP administration in healthy subjects did not result in pain sensitization including headache or somatic pain. Therefore, it seems that CGRP could not directly cause migraine pain and may mainly impose its effects via induction of different pathways such as stimulating the production of nitric oxide (NO), that finally could lead to neuro-inflammation and vasodilation which both might be involved in migraine pain [25, 36]. CGRP in combination with SP, PACAP, glutamate and neuropeptide Y could also induce a neurogenic inflammatory state in the CNS especially in trigeminovascular system. This inflammatory state is thought to be contributed to migraine pain sensation. Nonetheless, the activation of trigeminal pathway could also provoke the release of these factors and particularly might increase CGRP and SP secretion [9, 10, 25, 34, 35, 37–40].

To our knowledge this is the first investigation in migraineurs that assessed the levels of CGRP following vitamin D supplementation. According to available evidence, the mechanisms through which vitamin D influence CGRP synthesis and or release have not been clearly defined yet. There are a number of studies in which the effects of vitamin D or its derivatives on CGRP concentration were evaluated in animal models or cell cultures in different conditions; however, the findings have been inconclusive [41–43] due, in part, to the animal models applied or the dosage/duration of vitamin D administration.

In the present research, it was found that patients in the intervention group had significantly lower S25OHD levels, and on the other hand, a slightly higher serum CGRP levels at baseline; however, supplementation with the vitamin resulted in significant increment of S25OHD and reduction of CGRP, though we did not find any dose-response correlation throughout the trial between either of headache related parameters (e.g. frequency of headache days, MIDAS score or serum CGRP levels) and S25OHD. Therefore, it is likely that the protective effects of vitamin D in ameliorating migraine related features and CGRP levels reduction may be attributed to its anti-nociceptive properties and some indirect pathways. It is worth noting that NO, might elevate CGRP and SP synthesis/secretion release that ultimately may result in triggering nociceptive neurons and inflammation specially in trigeminovascular system [25, 34, 35, 39, 44]. On the flip side, decreased vitamin D concentration has been linked to elevated levels of NO. The protective efficacy of this vitamin against endothelial dysfunction as well as cardiovascular system could also be explained through this inverse relationship between NO and S5OHD levels [45]. Further, glutathione (GSH) presence in astrocytes may be associated with nitrogen and oxygen reactive species eliminating procedure (such as NO) [46, 47]. Since vitamin D active form (1,25(OH)2D3) may be involved in GSH metabolism, it can be speculated that vitamin D may also be involved in NO metabolism pathway [8].

### Study limitations

The strengths of this study are the prospective- double blind, placebo- controlled design, using a safe daily dosage of vitamin D as a somewhat inexpensive and available over-the-counter supplement, exploring CGRP levels as the dominant indicator of migraine pathogenesis, enrolling the study participant following examinations by expert headache-specialist neurologists, and confirming episodic migraine diagnosis based on the most recent ICHD criteria (ICHDIII). In spite of these strengths, there were a number of limitations. First of all, due to ethical considerations and according to migraine trials guidelines [27], it was not possible to discontinue the patients concomitant pharmacological therapy (including prophylactic or acute drugs). However, patients were recommended not to change their medications during the study. Also those subjects who received the medications that are supposed to interact with vitamin D were excluded from the trial. Second, although we tried to consider the effects of some confounding factors on the influences of vitamin D supplementation on headache outcomes through the ANCOVA, there might still be some other factors that could have been taken into account. Third, although both cases and controls were selected from the same population with similar characteristics including demographic, anthropometric and socioeconomic status, there could still be a chance of bias. Further, the current study was not powered enough to conduct a subgroup analysis between those with vitamin D deficiency/insufficiency. Thus, in future trials, subjects could be categorized according to S25OHD status so that appropriate dosage adjustment of vitamin D can be made. Additionally, investigating the efficacy of vitamin D on subgroups of migraine patients can be considered in the future trials including those with menstrual related migraine, chronic migraine or migraine with/without aura. Also, the effect of vitamin D receptor gene polymorphism should be clarified when studying the potential role of vitamin D supplementation in improvement of migraine characteristics and disability level.

## Conclusion

According to the current findings, vitamin D supplementation may potentially improve migraine headache, which has been established as the leading cause of disability in < 50 years, probably through attenuating CGRP levels. Therefore, daily administration of vitamin D 2000 IU as an adjuvant therapy could be considered a well-tolerated and effective agent in improvement of migraine characteristics and disability level particularly among those patients who have migraine with aura. This could provide a new insight into anti-nociceptive effects of vitamin D. However, to confirm current promising findings, large sample size RCTs with longer duration of follow-up are required especially in patients with chronic migraine. Also, well-designed animal and/or clinical studies addressing the mechanisms through which vitamin D may influence migraine pathogenesis, are of crucial importance.

## Data Availability

The datasets of the current study are available from the corresponding authors upon reasonable request.

## Abbreviations

ANCOVA: Analysis of covariance
cAMP: Cyclic adenosine monophosphate
CGRP: Calcitonin gene-related peptide
CI: Confidence interval
CLIA: Chemiluminescence immunoassays
CSD: Cortical spreading depression
ELIZA: Enzyme linked immunosorbent assay
EM: Episodic migraine
GBD: Global burden of disease
GSH: Glutathione
ICHDIII criteria: International Classification of Headache Disorders, 3rd edition
IL: Interleukin
iNOS: Inducible nitric oxide synthase
MIDAS: Migraine Disability Assessment Questionnaire
NO: Nitric oxide
PACAP: Pituitary adenylate cyclase-activating peptide
PNS and CNS: Peripheral and central nervous system
RCT: Randomized clinical trial
SD: Standard deviation
serum 25OHD: S25OHD
SP: Substance P
TNF-α: Tumor necrosis factor- α
